# Female Aging Affects Coilin Pattern in Mouse Cumulus Cells

**DOI:** 10.3390/jdb14010006

**Published:** 2026-01-15

**Authors:** Alexey S. Anisimov, Dmitry S. Bogolyubov, Irina O. Bogolyubova

**Affiliations:** 1 Department of Cytology and Histology, Faculty of Biology, Saint Petersburg State University, 199034 St. Petersburg, Russia; alexey.nisimov@gmail.com; 2 Institute of Cytology of the Russian Academy of Sciences, 194064 St. Petersburg, Russia; ibogol@mail.ru

**Keywords:** cumulus cells, granulosa cells, ovary aging, Cajal bodies, coilin, immunocytochemistry, bioinformatics analysis, 3D image analysis

## Abstract

Cumulus cells (CCs) are a distinct population of granulosa cells (GCs) that surround the developing and ovulated mammalian oocyte. The features of their structural organization and the expression pattern of key genes significantly affect oocyte viability. Changes in the functional activity of the nucleus are often expressed in changes in the structure of nuclear bodies (NBs), including Cajal bodies (CBs). The diagnostic protein of CBs is coilin, which maintains their structural integrity. Using fluorescent and electron microscopy, we examined maternal aging-associated changes in coilin pattern in mouse CCs. We found that older mice had a decrease in the number of coilin-positive bodies, while external transcriptome data analysis revealed no significant changes in *Coil* and *Smn1* gene expression. We hypothesized that the age-related dynamics of coilin-containing bodies are determined not by changes in the expression level of key components of these bodies, but by age-related changes in CC metabolism. Considering that CCs are a by-product of IVF protocols, making them available for analysis in sufficient quantities, age-related changes in the number and size of coilin-positive NBs in CCs may serve as a promising biomarker for assessing ovarian functional aging.

## 1. Introduction

One of the procedures aimed at providing women with infertility with the opportunity to have a child is in vitro fertilization (IVF) [1]. In the initial stages of this procedure, aspiration of the follicle contents is performed to obtain oocytes [2]. Along with the oocyte, cumulus cells (CCs) are extracted, which represent a subpopulation of granulosa cells (GCs) that are in direct contact with the oocyte and the cavity of the antral follicle [3].

The key role of CCs is to facilitate oocyte maturation. As the final cellular barrier between the oocyte and the environment, they perform vital trophic and regulatory functions that directly impact oocyte viability and its subsequent development [3]. Dysfunction of CCs is associated with a number of pathological conditions, such as polycystic ovary syndrome (PCOS) [4,5], GC tumor (GCT) [6,7], and premature menopause [8].

Although CCs are not used in standard IVF protocols, their genetic and biochemical characteristics may be proposed as biomarkers to predict oocyte development success [9]. However, the morphological features of CCs, including their nuclei, remain outside the scope of such studies, although nuclear compartmentalization and, primarily, the organization of the interchromatin space of the nucleus, can largely reflect the functional activity of the cell [10].

In this study, we analyzed the distribution of coilin protein in the CCs of mice of different ages. Although coilin is a predominantly nuclear protein and is difficult to detect in the cytoplasm by immunofluorescence microscopy or immunoblotting, it is nonetheless part of the nucleocytoplasmic transport system, shuttling between the nucleus and cytoplasm [11] and interacting with numerous proteins and RNA [12]. In the nucleus, the majority of the coilin molecule pool is diffusely distributed in the nucleoplasm, while the remainder is dynamically associated with specific nuclear bodies (NBs) [13]. The best-known NBs containing coilin are the Cajal bodies (CBs) [14]. These bodies are involved in many nuclear functions related to gene expression [15], the most important of which is the assembly/recycling of spliceosomal small nuclear (sn) RNPs [16]. It has long been known that coilin is a structural protein of CBs, acting as a kind of molecular “glue” that holds together the multiple protein and RNA components of CBs and thereby ensures the structural integrity of these nuclear bodies [12,17,18].

When assessing age-related changes at the cellular level, it is generally accepted that aging leads to disruption of cellular homeostasis, including changes in chromatin organization, transcriptional regulation, and NB composition [19,20,21]. In particular, CC aging leads to oxidative stress and metabolic disturbances in the cell [22].

In this context, CBs and CB-related NBs could likely serve as good indicators of these changes, as they are highly sensitive to specific genomic signals that modulate the nuclear molecular microenvironment [15]. For example, specific NBs have recently been discovered (in colorectal cancer cells) that are enriched in Ten-eleven translocation protein 1 (TET1)—an epigenetic regulator that initiates DNA demethylation as a result of a series of oxidative reactions, which causes an epigenetic imbalance in gene expression [23]. The authors noted that these TET1–NBs were 73% identical to CBs. In addition, CBs contain telomerase RNA (TR)—a key component, which increases the efficiency of telomerase holoenzyme assembly [24] and telomerase delivery to telomeres [25]. In turn, according to modern concepts, TR is involved in protecting cells from oxidative stress [26].

Three-dimensional segmentation and reconstruction algorithms are widely used to analyze nuclear architecture, including morphological features of various NBs [27]. We have developed an improved algorithm for the automatic analysis of a large number of cells [28]. This approach appears to be particularly important when studying highly dynamic NBs, where manual 3D analysis of hundreds of cells is difficult. Our tool automates the application of existing analysis methods to a large array of cells, representing an algorithm that controls the operation of other algorithms.

In this study, using our algorithm, we analyzed the morphometric characteristics of coilin-containing bodies in the nuclei of CCs obtained from mice of different ages, attempting to identify changes that may be associated with reproductive aging. To further interpret our data, we analyzed the transcriptome atlas of the aging mouse ovary [29], paying particular attention to age-related patterns of coilin expression.

## 2. Materials and Methods

### 2.1. Collection of Cumulus Cells

The study was performed on female BALB/c mice obtained from the “Rappolovo” nursery (Leningrad Region, Russia) of three age groups: 2–3 months (*n* = 3), 6–7 months (*n* = 3), and 12–14 months (*n* = 3). To synchronize the estrous cycle and increase the number of preovulatory follicles, ovarian stimulation was performed by a single intraperitoneal injection of 0.15 IU of pregnant mares’ serum gonadotropin “Folligon” (Intervet International B.V., Boxmeer, The Netherlands). Animals were sacrificed 48 h after injection by cervical dislocation, after which the ovaries were isolated and the follicle wall was punctured in modified HTF medium (Fujifilm Biosciences, Santa Ana, CA, USA) for air manipulations with the addition of 100 μg/mL dibutyryl cAMP (Sigma-Aldrich Co., St. Louis, MO, USA). Collected oocytes at the germinal vesicle (GV) stage were freed from CCs by pipetting. Next, CCs were collected and transferred to Polysine^®^ glass slides (Thermo scientific, Gerhard Menzel B.V. & Co. KG, Braunschweig, Germany).

### 2.2. Immunofluorescence Microscopy

Cells were fixed with 4% formaldehyde prepared from paraformaldehyde in PBS for 60 min at room temperature in a humid chamber. Permeabilization of cell membranes was performed for 10 min at room temperature in a 0.5% Triton X-100 solution, after which the preparations were incubated in 10% fetal serum (Gibco Laboratories, Grand Island, NY, USA) in PBS for 10 min at room temperature to prevent nonspecific binding of antibodies. The cells were then incubated with rabbit polyclonal antibody against coilin (Santa Cruz Biotechnology, sc-15408, Dallas, TX, USA) diluted 1:100 in 10% fetal serum for at least 12 h at 4 °C. After rinsing in PBS, cells were treated with Alexa 488-conjugated goat anti-rabbit antibody at a dilution of 1:200 for 75 min at room temperature. The preparations were embedded in Vectashield^®^ anti-fade medium (Vector Laboratories, Burlingame, CA, USA) containing 0.5 μg/mL DAPI. Preparations were analyzed in a Leica TCS SP5 laser scanning confocal microscope equipped with a set of appropriate lasers and 40×/1.25 objective. All digital images were obtained using identical settings. At least 30 nuclei were analyzed for each group.

### 2.3. Electron Microscopy

For conventional transmission electron microscopy (TEM), oocyte-cumulus complexes or ovarian pieces were fixed in 2.5% glutaraldehyde in 0.05 M cacodylate buffer, pH 7.3, then in 1.0% OsO_4_ in the same buffer and embedded in Spurr’s low viscosity medium (Electron Microscopy Sciences, Hatfield, PA, USA) according to manufacturer’s recommendations. Ultrathin sections were contrasted with uranyl acetate and lead citrate. For immunoelectron microscopy (IEM), the specimens were prepared as described [30]. Briefly, specimens were fixed for 2 h in 4% formaldehyde, freshly prepared from paraformaldehyde, containing 0.5% glutaraldehyde in PBS and then overnight in 2% formaldehyde at 4 °C. After rinsing in PBS containing 0.05 M NH_4_Cl and subsequent dehydration in an ethanol series, the specimens were embedded in LR White acrylic resin (Sigma-Aldrich Co., St. Louis, MO, USA). Ultrathin sections were incubated for 10 min in blocking buffer containing 0.5% fish gelatin and 0.02% Tween-20 in PBS, pH 7.4. After blocking, the sections were incubated in rabbit polyclonal serum against the carboxy-terminal fragment (14 kDa) of human coilin [31] overnight in a moist chamber at 4 °C. After rinsing in PBS containing 0.1% fish gelatin and 0.02% Tween-20, the sections were incubated with goat anti-rabbit antibody conjugated to 10 nm colloidal gold particles for 1.5 h in a moist chamber at room temperature. The sections were contrasted with uranyl acetate. The specimens were examined in a Leica 120 electron microscope (Carl Zeiss, Oberkochen, Germany) operated at 80 kV.

### 2.4. Quantitative Image Analysis

**Figure 1:**
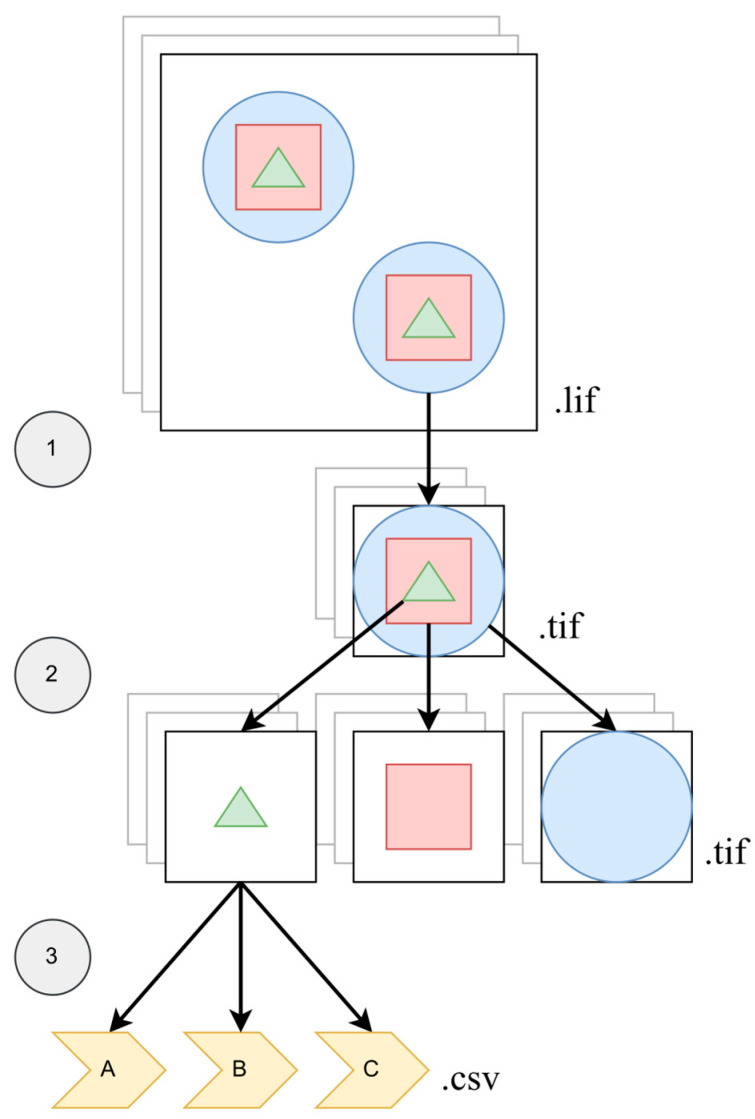
Schematic visualization of the macro generating data: (1) identification and cropping of individual nuclei from *.lif stacks; (2) segmentation of nuclear objects from both channels, generating segmented TIFF files; (3) extraction of quantitative parameters into tabular format (*.csv file). Nuclear objects are designated as follows: blue circle, nuclear contour; red square, DAPI-positive region; green triangle, localization zone of the protein of interest (coilin). A, B, C—the number of bodies, their size and fluorescence intensity.

For quantitative analysis of individual nuclei (*n* ≥ 30 per group), we developed an automated ImageJ (v. 2.17.0) macro and an R script pipeline (813 lines total; 57% ImageJ, 43% R). The source code is available in Zenodo [28]. The algorithm processes two-channel images—coilin (C1) and DAPI (C2). The pipeline comprises three modules (Figure 1): (i) identification and cropping of individual nuclei from *.lif stacks; (ii) segmentation of nuclear objects from both channels, generating segmented TIFF files; and (iii) extraction of quantitative parameters into tabular format (*.csv file).

Coilin-positive bodies were segmented using the following sequential parameters: 3D Gaussian blur (σ = 1 μm) followed by 3D maxima detection (3D ImageJ Suite [27]) with noise tolerance 0.25 and minimum distance 0.5 μm in XY and Z. For 3D segmentation, a threshold of 16 (0–256 a.u.) and a minimum object size of 0.1 μm were used. For the DAPI channel, segmentation of whole nuclei and DAPI-positive regions used a direct threshold-based approach without maxima detection, applying thresholds of 12 a.u. and 28 a.u., respectively. To ensure comparability of results between groups, all images were processed using the same iterative algorithm after parameter optimization. Coilin-positive bodies were identified in the vast majority of nuclei (90.7%). Quantitative data, including the number, size, and intensity of fluorescent staining of segmented objects, were obtained using the 3D ROI Manager plugin [27]. The parameters measured included the number of coilin-positive bodies in each nucleus and—for nuclei containing bodies—the mean quantitative characteristics (diameter and intensity) of each body per cell.

Quantitative metrics were optimized to ensure robustness given the limitations of fluorescence microscopy. Object diameter (μm) was calculated as the mean of the X- and Y-axis bounding box lengths, providing a consistent size estimate less susceptible to the influence of the lower axial resolution of confocal Z-stacks. The maximum gray value (a.u.) per 3D object was selected for signal intensity. This peak intensity provides a reliable measure of local protein density in punctate nuclear foci and is robust for comparing small, high-contrast structures [32].

To control data quality and reduce the influence of irrelevant factors on the final results, individual CCs/nuclei were excluded from further analysis according to the following criteria: (i) biological/methodological artifacts, namely the presence of abnormal structures, including contamination or mechanical damage to samples; (ii) the presence of overlapping cytoplasmic coilin-positive bodies, since the algorithm is focused exclusively on the quantitative analysis of nuclear spherical structures (Supplementary Figure S1).

### 2.5. Statistical Analysis

The calculation of modal values, as well as the testing of statistical hypotheses, were carried out using the R software environment (v. 4.5.0) (https://www.R-project.org/ accessed on 19 November 2025). Statistical significance was established at *p* < 0.05. Data distribution was assessed using the Shapiro–Wilk test (α = 0.05). Since the data deviated from normality, all results are presented as medians.

For categorical variables, such as the proportion of cells with significant cytoplasmic coilin labeling, associations were assessed using Fisher’s exact test (for small expected frequencies), followed by pairwise comparisons with Bonferroni correction.

For continuous parameters of coilin-positive bodies, we first tested pairwise differences between age groups of mice using Mann–Whitney tests with a Bonferroni correction. Parameters showing a pattern of differences consistent with an ordered trend were then analyzed using the Jonckheere–Terpstra test. The strength and direction of identified dependencies were quantified using the Kendall rank correlation coefficient (τ).

### 2.6. Analysis of External Transcriptome Data

Public single-cell RNA sequencing data (GEO: GSE232309) [29] was analyzed using Seurat (v. 5.3.0) [33] (https://satijalab.org/seurat/ accessed on 19 November 2025) in R software environment (v. 4.5.0). Differential expression analysis between antral GCs of aged (9-month) and young mice (3-month) was performed using the MAST framework (v. 1.33.0) [34] within Seurat’s FindMarkers function [33], selected for its robust handling of single-cell data characteristics including zero inflation. The analysis was performed on normalized data (layer = “data”) with pre-filtering thresholds, which requires detection of the gene in at least 30% of cells of one of the animal groups (min.pct = 0.3) and a minimal change in the expression level (logfc.threshold = 0.05). A significance threshold was defined as an adjusted *p*-value (*p*_adj_) < 0.05 combined with a minimum fold change of |log_2_FC| > 0.95 (approximately two-fold) to prioritize biologically substantial differences in expression. For functional characterization and gene description retrieval, differentially expressed genes were analyzed using the genome-wide annotation for mouse [35], followed by verification of functional associations using literary sources.

## 3. Results

### 3.1. Morphology and Distribution of Coilin-Containing Bodies

#### 3.1.1. Fluorescent Microscopy

A total of 144 CCs were analyzed, of which 15 (10.4%) were excluded from further analysis according to the established exclusion criteria (Section 2.4; Supplementary Figure S1). The final selection included 129 nuclei of CCs obtained from animals of different ages: 2–3 months (*n* = 54), 6–7 months (*n* = 33), and 12–14 months (*n* = 42).

**Figure 2:**
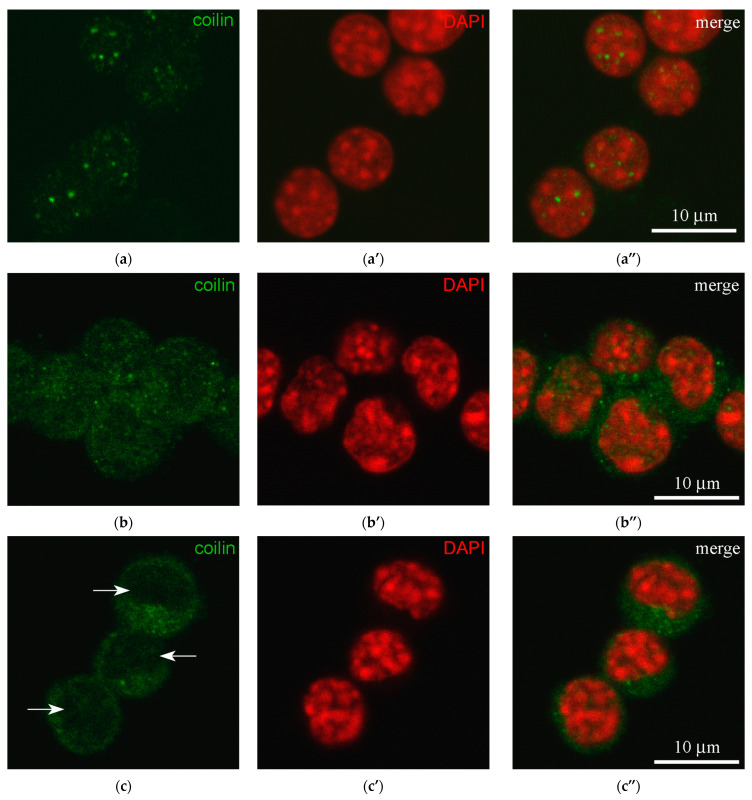
Three variants of coilin localization in mouse cumulus cells. (**a**–**a”**) Coilin is predominantly localized in the nucleus; (**b**–**b”**) Coilin is evenly distributed in both the nucleus and the cytoplasm, without noticeable foci. (**c**–**c”**) Coilin is predominantly localized in the cytoplasm; arrows point to unstained nuclei. (**a**–**c**) Antibody staining < coilin is green; (**a’**–**c’**) DAPI staining (artificial red); (**a”**–**c”**) Merged images.

Coilin signal was observed in the vast majority of nuclei analyzed (Figure 2a–a”), and the cytoplasm was unstained in most cells (89.1%). However, some individual cells with pronounced cytoplasmic coilin labeling were also observed (Figure 2b–b”). This pattern of coilin distribution, including rare cytoplasmic labeling, was found in 16.7% of CCs in young animals (9 of 54) and in 15.2% of cells in middle-aged mice (5 of 33). In the group of old mice (12–14 months), coilin labeling of the cytoplasm was not observed (0 of 42). In addition, four cells were found (1 and 3 in the young and middle-aged groups of animals, respectively) in which coilin-positive foci in the nuclei were absent, despite a pronounced coilin signal in the cytoplasm (Figure 2c–c”).

Fisher’s exact test revealed statistically significant differences between groups in the presence of coilin in the cytoplasm (*p* = 0.00866). Subsequent pairwise comparisons confirmed that in the older age group of mice (12–14 months), the proportion of CCs with coilin detected in the cytoplasm was significantly different (*p*_adj_ < 0.05) from the other two groups (2–3 months and 6–7 months), whereas the differences between animals of the younger and middle age groups were not significant.

**Figure 3:**
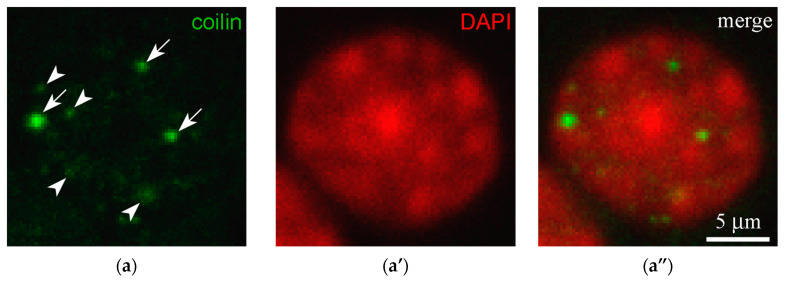
Confocal images showing the typical distribution pattern of coilin in the nuclei of mouse cumulus cells, demonstrating larger (arrows) and smaller foci (arrowheads) in the nucleus. (**a**) Coilin staining (green); (**a’**) DAPI staining (artificial red); (**a”**) merged image.

A typical pattern of chromatin and coilin in the nuclei of CCs is shown in Figure 3 (see also Supplementary Figure S2 for other mammalian somatic cells). Coilin-positive foci were represented by both relatively large (Figure 3, arrows) and smaller, blurry entities (Figure 3, arrowheads). The latter had lower fluorescent staining intensity.

#### 3.1.2. Electron Microscopy

**Figure 4:**
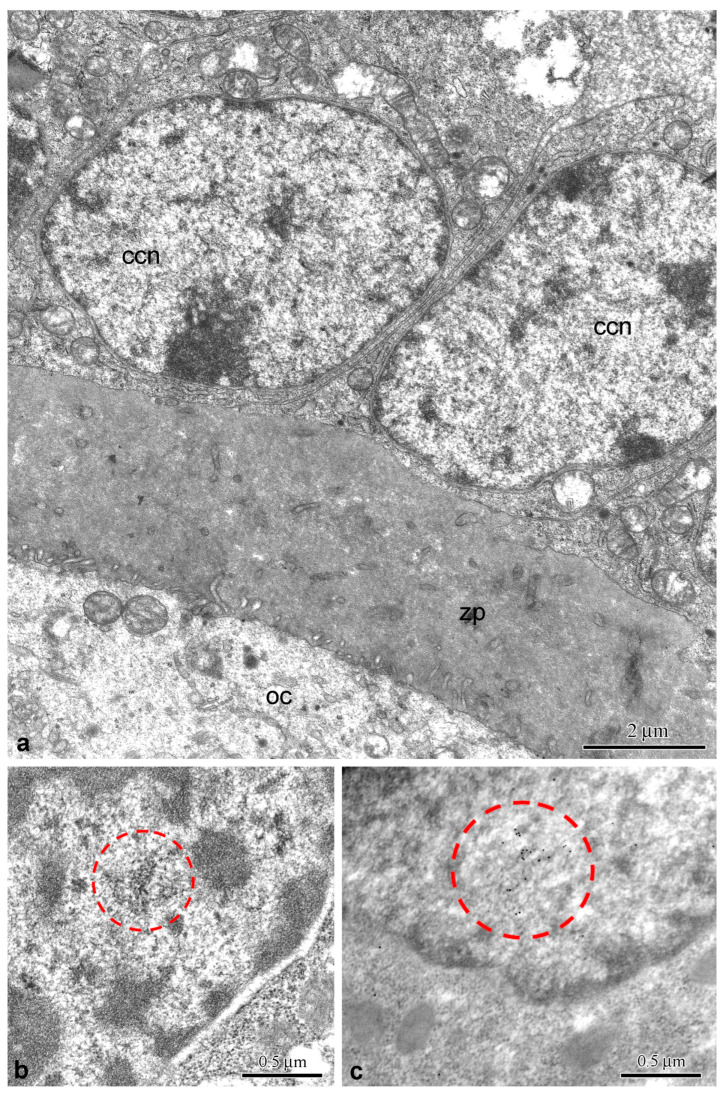
Ultrastructure of mouse cumulus cell nuclei. (**a**) General view; ccn, cumulus cell nucleus; oc, oocyte; zp, *zona pellucida*. (**b**) A Cajal body-like structure (outlined) composed of coiled threads, seen at higher magnification. (**c**) Immunogold labeling with coilin antibody, demonstrating the concentration of labels above the body (outlined).

Ultrastructural studies have shown that the nuclei of CCs contain small formations not exceeding 0.5 μm, the structural details of which can only be seen at higher magnification (Figure 4a,b). They consist of characteristic coiled threads, 30–40 nm in thickness (Figure 4b), which correspond to the elementary morphological structures of canonical CBs of mammalian somatic cells [36]. The very loose packing of such coiled threads is noteworthy, which sometimes makes it difficult to morphologically distinguish these CB-like formations from the surrounding nucleoplasmic structures. This feature somewhat distinguishes the putative CBs observed in mouse CCs from typical/canonical mammalian somatic cell CBs. In the latter case, CBs are electron-dense, more compact NBs consisting of tightly packed coiled threads (Supplementary Figure S3). No differences in the ultrastructure of presumptive CBs in CCs were detected in young and old mice. Treatment of ultrathin sections with coilin antibody (IEM) revealed a concentration of labeling over these structures (Figure 4c), confirming that they are related to CBs. However, the fine morphology of these “bodies” was poorly preserved during the preparation of samples for IEM. Some coilin labels were observed both in the nucleoplasm and the cytoplasm.

### 3.2. Age-Related Dynamics of Coilin Bodies

Quantitative assessment of age-related differences in the morphological characteristics of nuclear coilin-positive fluorescent foci/bodies (Section 3.1.1) was performed using special macro scripts (Section 2.4). Processing of 18 digital images allowed the identification of 129 nuclei, which was fully consistent with the results of manual analysis.

**Figure 5:**
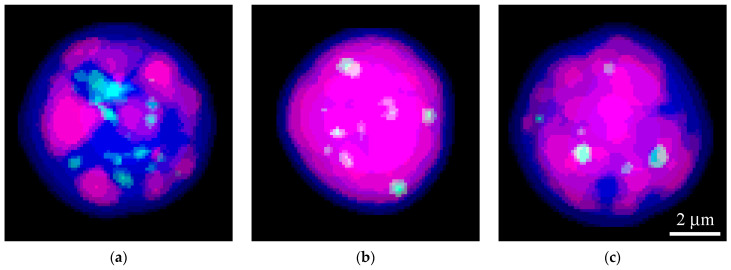
Representative images of coilin-positive bodies (green) for each age group of mice: (**a**) 2–3 months, (**b**) 6–7 months, (**c**) 12–14 months. The nuclei are segmented according to [27]. Nuclear contours are visualized by DAPI staining (blue), where high-intensity signal areas (red) correspond to regions enriched in AT repeats.

Visual inspection of nuclear morphology revealed a marked age-related decrease in the number of coilin-positive foci, apparently corresponding to coilin-containing NBs. To quantify this phenomenon, we applied our segmentation algorithm, identifying a total of 813 coilin-positive bodies across all analyzed nuclei: 439 in young, 210 in middle-aged, and 164 in old animals (Figure 5). The distribution of data on the number, volume and intensity of these bodies deviated from the norm for all groups (Shapiro–Wilk test, *p* < 0.05), which required the use of nonparametric analysis.

**Figure 6:**
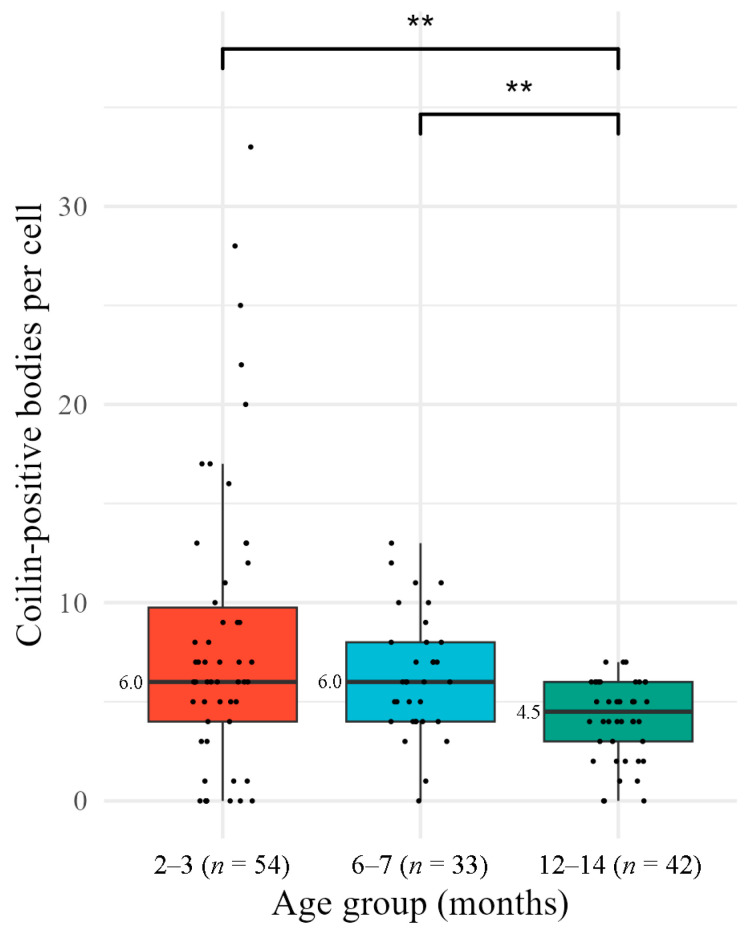
The number of coilin-positive foci/bodies per cumulus cell in different age groups of mice. Box plots show the median and interquartile range (IQR). Individual data points (each representing one nucleus) are overlaid as dots. Colors correspond to mouse age groups: red for 2–3 months, blue for 6–7 months, green for 12–14 months; **—differences between groups are significant, *p* < 0.01; *n*, number of analyzed nuclei.

The median values of the number of coilin-positive foci/bodies for young and middle-aged animals were 6 per nucleus. In the group of old animals, the median value was significantly lower (Figure 6). When analyzing the number of coilin-positive bodies in the nuclei, an asymmetric distribution of data was observed with a shift towards lower values, while rare extreme values were recorded in the group of young animals. In particular, in young animals, the number of coilin-positive bodies in individual nuclei reached 33, which is 2.5 times higher than the maximum value in middle-aged mice (13) and 4.7 times higher than the maximum value in old individuals (7).

Comparison of the studied groups of mice revealed significant differences between young (2–3 months) and old (12–14 months) animals (Mann–Whitney test: U = 1560, *p* = 0.0047), as well as between 6–7- and 12–14-month groups (U = 987, *p* = 0.0047). However, differences between the younger and middle-aged groups were not significant (*p* > 0.05).

The Jonckheere test (JT) has demonstrated the presence of a statistically significant ordered trend of decreasing number of coilin-positive bodies with increasing age (*z* = −3.38, *p* = 0.0007). Although Kendall’s τ correlation coefficient indicated a weak inverse relationship (*τ* = −0.241), its statistical significance (*p* = 0.0007) confirms the existence of biologically relevant age-related dynamics.

**Figure 7:**
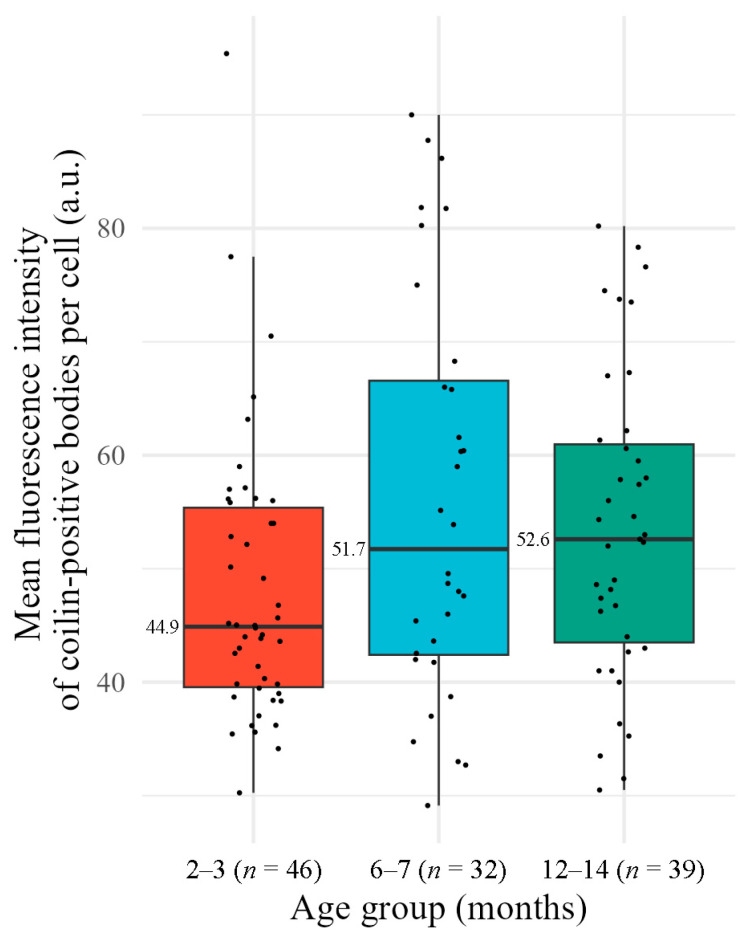
Mean fluorescence intensity of coilin-positive bodies per cumulus cell in different age groups of mice. Box plots show the median and interquartile range (IQR). Individual data points (each representing one nucleus) are overlaid as dots. Colors correspond to mouse age groups: red for 2–3 months, blue for 6–7 months, green for 12–14 months; *n*, number of analyzed nuclei.

For nuclei containing at least one coilin-positive body, we analyzed fluorescence intensity as maximum gray value (a.u.) using the 3D ROI Manager plugin [27]. The median intensity per cell was similar across age groups (Figure 7). Pairwise comparisons using Mann–Whitney tests with Bonferroni correction revealed no statistically significant differences between the groups (*p* > 0.05). Given the lack of significant intergroup differences, trend analysis was not performed.

**Figure 8:**
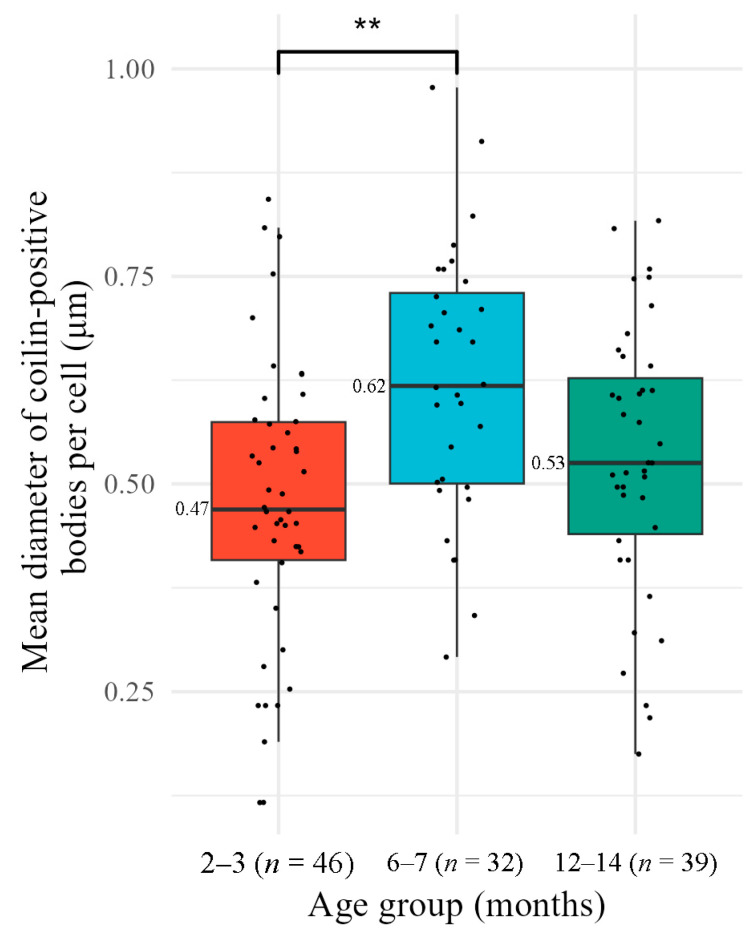
Mean diameter of coilin-positive bodies per cumulus cell in different age groups of mice. Box plots show the median and interquartile range (IQR). Individual data points (each representing one nucleus) are overlaid as dots. Colors correspond to mouse age groups: red for 2–3 months, blue for 6–7 months, green for 12–14 months; **—difference is significant, *p* < 0.01; *n*, number of analyzed nuclei.

Fluorescence microscopy has shown that pronounced foci of coilin are not present in all CC nuclei (Section 3.1.1). In those nuclei where these foci were present, their mean diameter per cell was calculated from the XY bounding boxes (Figure 8). Pairwise comparisons revealed a statistically significant difference between 2–3-month and 6–7-month groups (Mann–Whitney test, *p* = 0.0018). However, this apparent increase of 0.15 µm is below the XY diffraction limit of our imaging system (~0.26 µm for Alexa 488 with a 40×/1.25 NA objective). Furthermore, the Jonckheere–Terpstra test did not reveal a significant ordered trend across all age groups. (JT = 2588, *p* = 0.096). Given this, an isolated pairwise difference does not represent a biologically significant age-related change in coilin-containing body size.

### 3.3. Re-Analysis of Published Transcriptome Data

To contextualize our immunocytochemical findings on coilin-positive bodies within broader transcriptional changes in aging ovarian cells, we first re-analyzed a publicly available single-cell RNA sequencing dataset [29]. The authors provided a pre-processed GC transcriptome that had already been isolated from the complete ovarian dataset, containing 4 subpopulations of GCs: antral, preantral, mitotic, and atretic. After reproducing their GC subclustering and marker gene expression patterns, we focused specifically on the antral GC subcluster, as its CC content had not been clearly characterized.

**Figure 9:**
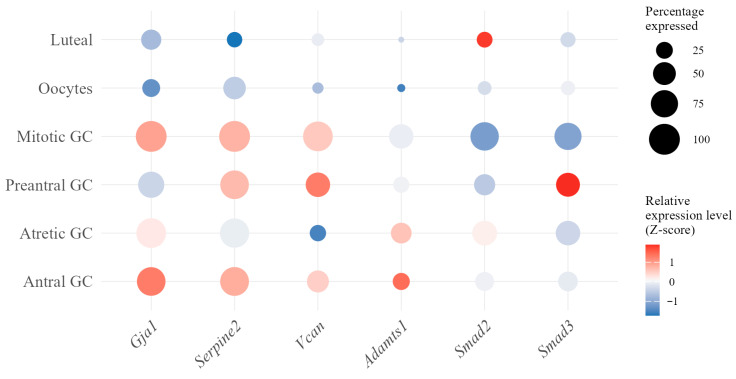
Cumulus cell marker expression across granulosa subpopulations. Antral granulosa cells (GCs) show strongest expression of *Gja1*, *Serpine2*, *Adamts1*, and *Vcan*, confirming that they belong to the cumulus-containing subpopulation. Color intensity represents Z-scored expression; dot size indicates the percentage of cells expressing the gene (percentage expressed).

To verify that the antral GC subpopulation contained CCs relevant to our experimental model, we analyzed *Gja1* encoding connexin 43—a critical gap junction component essential for cumulus-oocyte communication [37,38]—and *Serpine2*, a product of which mediates extracellular matrix remodeling during cumulus expansion [38]. Additionally, we examined extracellular matrix genes *Vcan* and *Adamts1* [39,40], as well as the *Smad2* and *Smad3* genes, which encode signaling components that regulate CC functions [41]. Our analysis confirmed that antral GCs exhibit the strongest cumulus signature, with 4 of 6 markers highly expressed (*Gja1*: *Z*-score = 1.28 in 84% of cells; *Serpine2*: *Z*-score = 0.83 in 85% of cells; *Adamts1*: *Z*-score = 1.42; *Vcan*: *Z*-score = 0.48) (Figure 9).

Critically, unlike mitotic GCs that show dramatic downregulation of *Smad2/3* (*Z*-scores: −1.15, −1.08), antral cells maintain normal *Smad2/3* signaling compared to all granulosa clusters. This validation confirms that the antral granulosa subcluster represents the most appropriate transcriptomic reference for our population, as it not only matches our experimental source (CCs from mature antral follicles) but also demonstrates expression patterns consistent with established CC markers in the literature [37,38,39,40,41].

Using MAST analysis optimized for single-cell data [34], we identified aging-associated transcriptional changes that extend beyond the pathway-level alterations reported by Isola et al. [29]. Their use of Ingenuity Pathway Analysis (IPA) method allowed them to identify fibrosis (hepatic fibrosis signaling) and oxidative stress (oxidative phosphorylation) as the main changes in antral GCs associated with aging [29].

Our gene-level analysis validated these findings, revealing significant downregulation of cytoskeleton and cell adhesion genes with established roles in fibrotic processes. While ovarian fibrosis is a well-documented phenomenon primarily affecting the stromal compartment [42], we found a downregulation of a distinct set of genes with established roles in fibrotic pathways in CCs, including *Thbs1* [43] (log_2_FC = −1.85), *Unc5c* [44] (−1.82), *Lrrc4* [45] (−1.54), *Tmsb4x* [46] (−0.97), *Epha4* [47] (−0.96), and *Dock4* [48] (−0.96). We similarly confirmed oxidative stress response patterns with upregulation of glutathione pathway components *Gstm7* (log_2_FC = 1.52), *Gsta4* (1.42), *Gstp1* (1.00) [49,50], and stress response regulators *Ddit4* (1.68) and *Tusc2* (1.16) [51,52].

**Figure 10:**
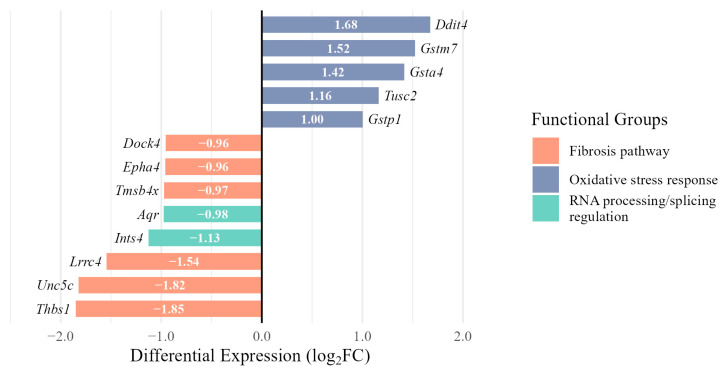
Differential expression of some key genes involved in fibrosis, oxidative stress response, and RNA processing/splicing regulation in antral granulosa cells from 9-month-old compared to 3-month-old mice.

In addition to these confirmed signaling pathways, our analysis revealed a specific and significant reduction in the expression of two genes that are involved in the regulation of pre-mRNA splicing. These are *Aquarius* (*Aqr*) involved in pre-mRNA splicing via spliceosomes [53] and *Ints4* encoding one of the catalytic subunits of the Integrator complex, responsible for 3′ end processing of spliceosomal pre-snRNA [54]. These genes showed approximately a 2-fold decrease in expression (log_2_FC = −0.98 and −1.13, respectively). This finding is particularly relevant given the key role of CBs in the biogenesis of spliceosome components [16,55,56]. Differential expression of some representative key genes related to the fibrosis pathway, oxidative stress response, and RNA processing/splicing regulation (*Aqr* and *Ints4*) is presented as a bar plot (Figure 10). An expanded list of genes differentially expressed in antral GCs is presented in Supplementary Materials (Tables S1 and S2).

Interestingly, the *Smn1* gene encoding the SMN protein—a key and best-known coilin interactor [57] that serves as the main “master assembler” of RNP particles including spliceosomal snRNPs [58], showed a minimal change (log_2_FC = 0.40). At the same time, the expression level of the coilin gene *Coil* [59] did not undergo significant changes (*p*_adj_ > 0.05).

## 4. Discussion

Cumulus cells (CCs) are a specialized subpopulation of GCs that supports the growth and maturation of mammalian oocytes in follicles [60]. In this study, we found that the pattern of coilin distribution and the quantitative characteristics of coilin-positive NBs differ in CCs obtained from animals of different ages. The most noticeable change was a decrease in the number of coilin-positive bodies in the CCs of older animals.

We used three age groups of mice: 2–3 months, 6–7 months, and 12–14 months. According to approximate mouse/human age comparisons presented by Flurkey et al. [61], these age groups for C57BL/6J mice correspond approximately to 18–20, 30–32, and 42.5–47 years in humans, respectively. In the present study, we used another mouse strain—BALB/c—so these age correlations may be somewhat different due to the lifespan differences between mice of various strains [62]. Nevertheless, we consider old mice as a model of pronounced age-related decline in fertility, especially compared to young animals (2–3 months), since clinical studies have shown a marked decline in fertility in women over 42 years of age [63]. Animals aged 6–7 months approximately correspond to young women (<35 years) with relatively high fertility [63,64].

The features of the coilin pattern that we identified, characteristic of older animals, can be considered as associated with an age-related decline in fertility. However, it cannot be ruled out that age-associated changes in CCs may outpace the overall decline in fertility and reflect individual characteristics of biological aging in the female body. This highlights the potential importance of such changes for predicting possible IVF outcomes.

Our ultrastructural study unexpectedly did not reveal any noticeable coilin-containing structures that could be called NB in the strict sense of the word [65], although they could be identified morphologically by the presence of specific coiled threads [36] characteristic of the CBs [14]. Regardless of the age of the mice, these structures were only highly loosened snarls or tangles of such threads, poorly distinguishable from the surrounding nucleoplasm (Figure 4 and Supplementary Figure S3). When using antibodies to coilin after fixation with formaldehyde and embedding in LR White resin, their fine structure was even less visible. This situation is somewhat reminiscent of the one with the so-called Polycomb bodies [66]. In fact, electron microscopy failed to detect these “bodies” as such. However, their marker—the Polycomb group (PcG) protein BMI1—showed localized concentrations in specific nuclear regions that were expected to correspond to PcG bodies based on fluorescence microscopy [67].

Coilin—a scaffold protein of CBs—is not an unambiguous diagnostic marker for this type of NBs, as it also marks some histone locus bodies (HLBs). While in *Drosophila* or *Xenopus* all HLBs appear to contain coilin, in zebrafish *Danio rerio* the components of histone pre-mRNA 3′-end processing are localized exclusively in coilin-negative HLBs [68,69]. In this case, coilin specifically marks the CBs. In contrast, a significant proportion of HLBs in human cells contain coilin [70,71]. Herein, we did not perform further identification of coilin-positive bodies that we observed in the nuclei of mouse CCs.

Although HLBs per se are not a necessary attribute of histone pre-mRNA processing, they accumulate unique factors of replication-dependent (RD) transcription and histone pre-mRNA processing, thereby intensifying these processes in the S phase of the cell cycle [72]. Experimental destruction of HLBs leads to a significant decrease in the intensity of RD transcription of histone genes and cell cycle arrest in the S phase [73]. Follicular CCs are proliferating cells, and about 30% of them are in S phase. Only after ovulation are they predominantly in the G0/G1 phase, while S-phase cells are relatively absent in the oocyte-cumulus complexes [74]. Therefore, we believe that at least a portion of the coilin-containing NBs of CCs may likely represent HLBs. The presence of HLB and its dynamics in CCs have not yet been demonstrated.

The pattern of coilin localization in the cell nucleus and the CBs themselves is highly dynamic [75]. It significantly depends on the cell’s functional state, including coilin phosphorylation [76], overall transcriptional activity of the nucleus, and the expression level of genes encoding CB structural proteins [77], as well as the cell cycle stage [15] and various stresses [78].

Thus, the dynamics of coilin-containing bodies may reflect changes in the transcriptional and/or metabolic activity of the cell, since it is generally accepted that CBs are involved in the assembly/recycling of spliceosomal snRNP particles and scaRNA-directed modifications of splicing snRNAs. In particular, CBs are associated with the formation of functionally competent snRNP subunits of spliceosomes [16], although pre-mRNA splicing itself does not occur in them [79]. SnoRNAs of different classes, including scaRNAs that specifically modify snRNA molecules, necessarily pass through CBs [80,81]. The presence of CBs near transcribed genes leads to the creation of specific intergenic and/or interchromosomal regulatory hubs, which, in turn, can serve as the nucleation sites for specific nuclear organelles, including CBs [82]. The appearance of such hubs enhances nuclear processes associated with transcription and splicing, and simultaneously reflects the intensification of the corresponding processes [83].

It has long been known that CBs often form in close proximity to gene clusters encoding major and minor snRNAs, such as U1, U2, U4, U11, and U12, but not U6 snRNAs [84,85]. The close relationship between CBs and snRNA genes was clearly demonstrable by chromatin immunoprecipitation experiments followed by DNA sequencing [80]. Apparently, CBs can directly regulate snRNA synthesis in a feedback manner, being sensitive to the total amount of snRNA in the nucleus [86], since primary snRNA transcripts (e.g., U2 pre-snRNA) mediate the association of CBs with snRNA genes [87].

Given the complexity and multifactorality of signaling pathways in CCs, as well as the presence of multiple coding and noncoding transcripts [88], it is impossible to assume that aging is accompanied by a simple decrease in the transcriptional activity of the nuclei of these cells. Our findings of altered distribution patterns of coilin-containing NBs are consistent with this, suggesting these changes are associated with changes in transcription patterns but not with a significant decrease in the overall level of transcriptional activity in CCs of aged mice. Conversely, since at least a significant proportion of coilin-containing bodies are likely to be CBs, one would expect a sharp decrease in their number or complete disappearance if CC aging were accompanied by a decrease in overall transcription.

Although as females aged, we observed a significant decrease in the number of coilin-containing bodies, their median number in the CCs of old mice was 4.5 per nucleus, which corresponds to the average number of CBs in somatic cells of mammals [89]. However, in this regard, it is worth noting that in some cells whose metabolic activity is significantly reduced under certain physiological conditions, noticeable NBs containing coilin and some other CB components can be detected [90], but these are not canonical CBs [91].

To further interpret our morphological data, we analyzed age-associated changes in the transcriptional profile of antral GCs using a published database [29]. Here, we did not cluster CCs among the overall antral GC population but hypothesized that significant transcriptomic changes in antral GCs are reflected in CCs (Figure 9). In other words, antral GCs are largely composed of CCs and are their representative transcriptome model.

Importantly, neither the *Coil* nor the *Smn1* gene showed significant changes in their expression in antral GCs, suggesting that age-related disorganization of coilin-positive bodies in mouse CCs likely occurs through mechanisms other than decreased transcription of the essential CB components—coilin and SMN. For example, the age-related dynamics of coilin-containing NBs could be due to a decrease in the expression of some factors involved in the formation and functioning of spliceosomes. One can speculate that the marked decrease in the number of CBs in CCs of old mice may be due, for example, to a decrease in the expression of the genes involved in RNA processing.

One of such candidates may be the *Aquarius* (*Aqr*) gene, which encodes a protein that plays a key role in the biogenesis of C/D box sno/scaRNAs and in pre-mRNA splicing [53,92]. The Aquarius protein is an RNA helicase that is integrated into spliceosomes, directly interacts with U2 snRNP proteins, and binds pre-mRNA introns during splicing [93]. Another candidate may be *Ints4* encoding one of the catalytic subunits of the Integrator complex, which associates with the C-terminal domain of RNA polymerase II and mediates 3′ end processing of U1 and U2 snRNAs [54,94]—key RNA components of the major spliceosomes. It has been shown that deficiency of INTC4 leads to the destruction of both CBs and HLBs [95,96]. However, the proposed role of downregulation of *Aqr* and *Ints4* remains associative rather than causal.

According to established concepts, reproductive aging is associated with a significant redox imbalance in the oocyte microenvironment inside the follicle, in other words, with a high oxidative stress [62,97] This imbalance promotes cellular damage, as supported by direct evidence of increased levels of apoptotic markers in CCs [98] and upregulation of stress response signaling pathways [29]. This will inevitably lead to a change in the chemical composition of both the cytoplasm and the nucleoplasm, possibly affecting the dynamics of self-assembly of coilin-containing bodies.

In general, all NBs are membraneless organelles formed as a result of liquid–liquid phase separation (LLPS). This process is highly sensitive to environmental conditions, such as various stresses, including UV radiation, ATP depletion, oxidative stress, pH changes, and others [99]. To maintain their biological functions, nuclear biomolecular condensates (NBs) typically have a fluid-like consistency, but their maturation or aging leads to a more rigid aggregated state. It is frequently associated with neurodegenerative diseases [99]. However, it cannot be ruled out that similar changes may also occur with coilin-containing NBs in the nuclei of aging cumulus cells.

In any case, the nature, molecular composition and biological significance of coilin-containing NBs in CCs remain to be determined, as do the mechanisms of regulation of oogenesis by CCs, which involve various nuclear molecular machinery. At the same time, the identified age-related changes in the number and size of coilin-positive bodies in CCs suggest that these parameters may serve as a promising biomarker for assessing ovarian functional aging. Given that CCs are a by-product of IVF protocols, making them available for analysis in sufficient quantities, further research in this area may facilitate the development of new tests to assess the extent of age-related changes in oocyte-cumulus complexes.

Limitations: In accordance with the 3R concept, we reduced the number of animals used to 3 females per group. The small sample size may affect the generalizability of the results. However, taking into account the sufficient number of nuclei analyzed (at least 30 per group), as well as the use of appropriate statistical analysis methods, we believe that this influence is minimal and the data we obtained are reliable.

## 5. Conclusions

Coilin distribution pattern differs in CCs of mice of different ages. This is primarily manifested in a gradual decrease in the number of coilin-positive NBs in older animals, which is not accompanied by significant changes in the expression of either the *Coil* or *Smn1* genes based on analysis of external transcriptome data. At present, it is difficult to provide a clear mechanistic explanation for the age-related dynamics of the coilin pattern in CCs. However, regardless of the factors causing these rearrangements, coilin-positive and possibly other NBs of CCs require further study in the search for potential biomarkers reflecting individual rates of ovarian aging.

## Data Availability

The data presented in this study are available on request from the corresponding author.

## Supplementary Materials

The following supporting information can be downloaded at: https://www.mdpi.com/article/10.3390/jdb14010006/s1, Figure S1: Examples of Cumulus Cell Nuclei Excluded from Analysis; Figure S2: Typical Distribution of Coilin in Mammalian Cell Nuclei; Figure S3: Ultrastructure of Mouse Cajal Bodies; Differentially Expressed Genes in Mouse Antral Granulosa Cells, Table S1: Downregulated genes; Table S2: Upregulated genes.

## Abbreviations

The following abbreviations are used in this manuscript:

CB	Cajal body
CC	Cumulus cell
GC	Granulosa cell
HLB	Histone locus body
IEM	Immunogold/electron microscopy
IVF	In vitro fertilization
LLPS	Liquid–liquid phase separation
NB	Nuclear body
PBS	Phosphate-buffered saline
TEM	Transmission electron microscopy
