# Supplementary material for: Female Aging Affects Coilin Pattern in Mouse Cumulus Cells
Source: J Dev Biol. 2026 Jan 15;14(1):6. doi: 10.3390/jdb14010006 (PMC12821526; doi:10.3390/jdb14010006)
Supplement: Supplementary file 1 [file jdb-14-00006-s001.zip › jdb-4042570-supplementary.pdf]

Supplementary Materials

Suppl. S1. Examples of Cumulus Cell Nuclei Excluded from Analysis

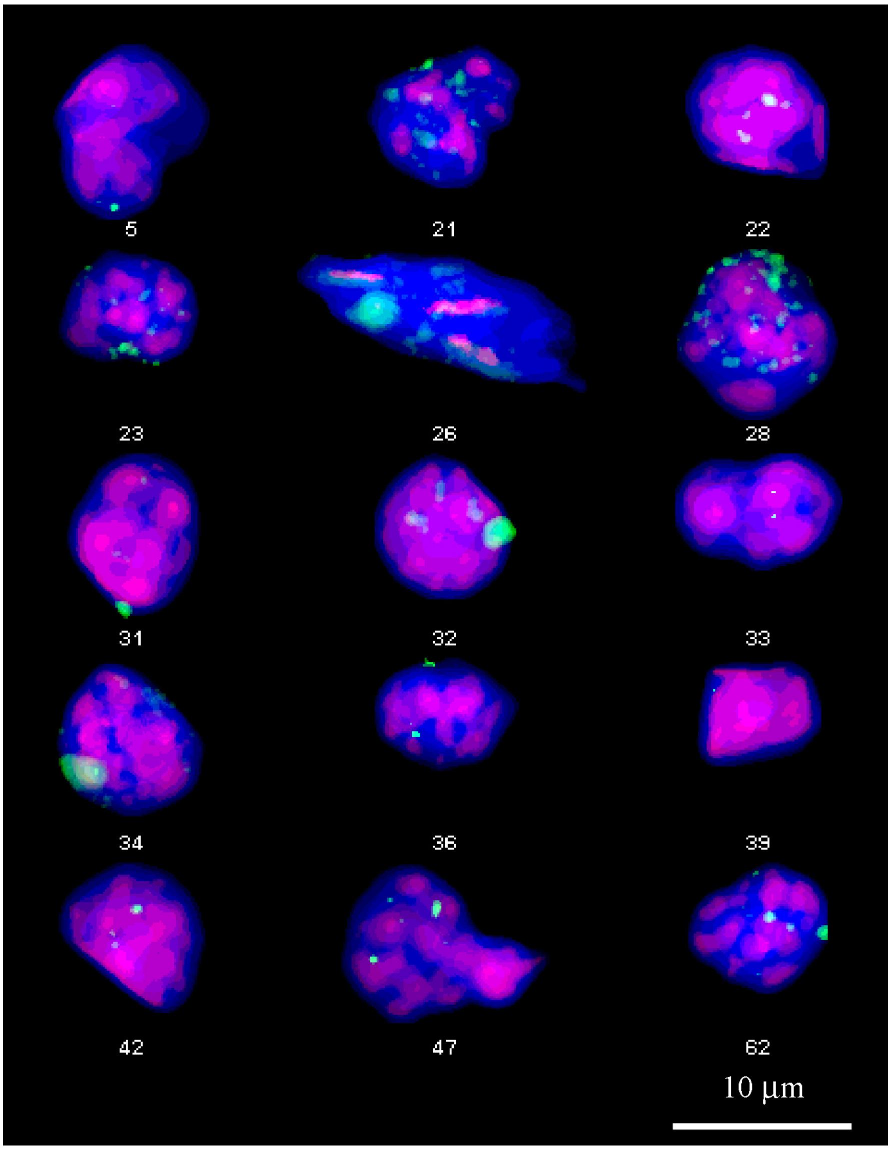

Figure S1. Depiction of all Mouse cumulus cell nuclei (n=15) excluded from our primary dataset of 144 nuclei. Reasons for exclusion were based on biological/methodological artifacts, namely the presence of contamination (## 32, 34), mechanical damage to the samples (# 26), strongly irregular nucleus shape (## 5, 21, 22, 33, 39, 42, 47), or demonstrated the presence of overlapping cytoplasmic coilin-positive formations (## 23, 28, 31, 36, 62). The numbers correspond to the original identification numbers assigned to each nucleus during initial analysis for tracking purposes.

Suppl. S2. Typical Distribution of Coilin in Mammalian Cell Nuclei

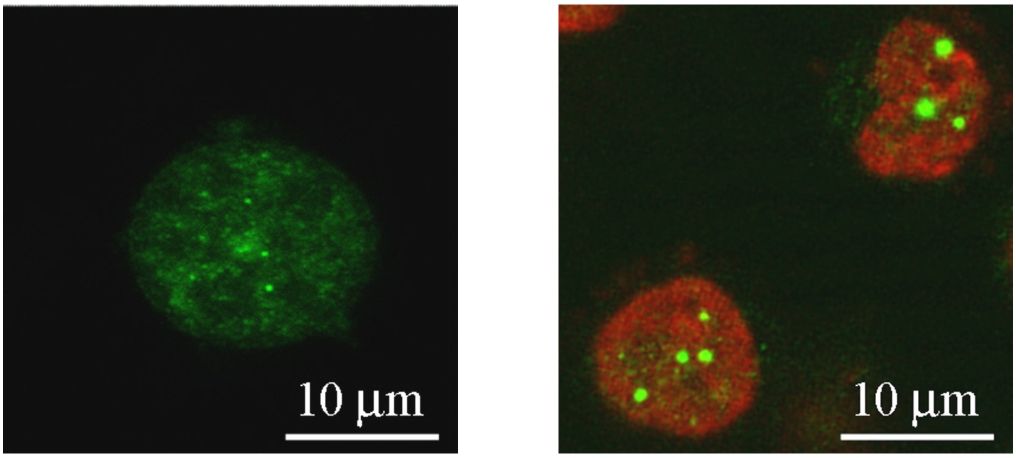

(**a**) (**b**)

Figure S2. Coilin distribution in the nuclei of mouse hepatocyte (**a**) and human cells (HeLa) (**b**). Coilin is green; in (b), nuclei are counterstained with DAPI (artificial red). The overall patterns are generally consistent with the distribution of coilin in the nuclei of mouse cumulus cells (see Figure 2 in the main text), although the size of coilin foci may vary and is not related to the size of the nucleus itself.

Suppl. S3. Ultrastructure of Mouse Cajal Bodies

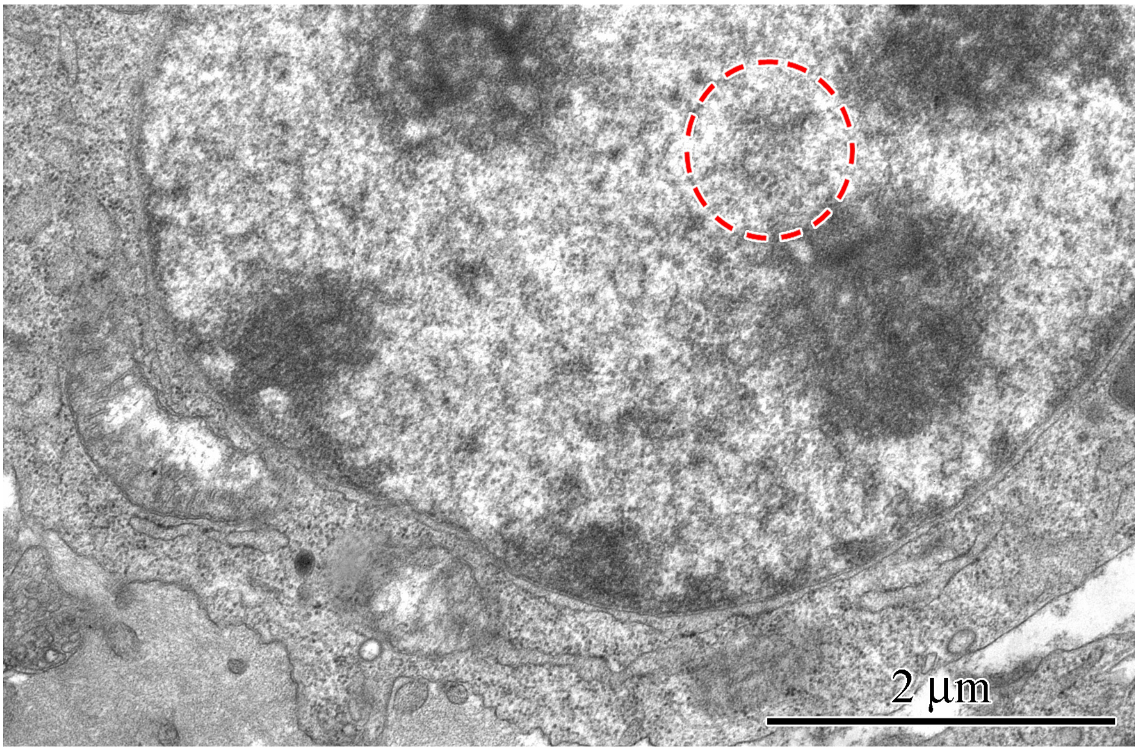

(**a**)

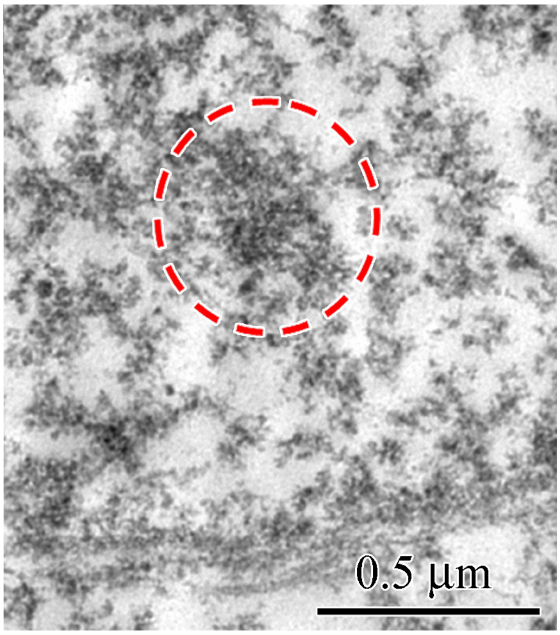

(**b**)

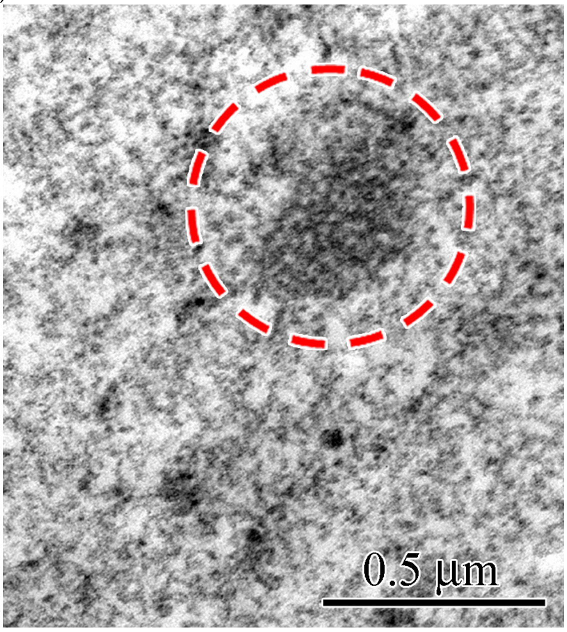

(**c**)

Figure S3. Ultrastructure of mouse Cajal bodies (encircled). (**a**) Low-magnification fragment of a cumulus cell nucleus showing the putative Cajal body poorly distinguishable from the surrounding nucleoplasm. (**b**) Similar structure in a cumulus cell nucleus at higher magnification; see also Figure 4b in the main text. (**c**) A typical Cajal body in the nucleus of a mouse hepatocyte, consisting of 30-nm coiled threads packed more densely than the putative Cajal body in cumulus cells.

Suppl. S4. Differentially Expressed Genes in Mouse Antral Granulosa Cells

Table S1. Downregulated genes.

Gene Symbol	Log_2 Fold Change	Protein Name
Thbs1	–1.8515532	thrombospondin 1
Unc5c	–1.8211288	unc–5 netrin receptor C
Hecw2	–1.4731860	HECT, C2 and WW domain containing E3 ubiquitin protein ligase 2
Pygm	–1.4646277	muscle glycogen phosphorylase
Clstn2	–1.4291752	calsyntenin 2
Kirrel3	–1.3874009	kirre like nephrin family adhesion molecule 3
Naa25	–1.3359032	N(alpha)–acetyltransferase 25, NatB auxiliary subunit
Ank3	–1.3033909	ankyrin 3, epithelial
Pycard	–1.2615293	PYD and CARD domain containing
Slc24a5	–1.2463732	solute carrier family 24, member 5
Ppp4r1	–1.1942258	protein phosphatase 4, regulatory subunit 1
Usp6nl	–1.1906282	USP6 N–terminal like
Ldlrad4	–1.1858768	low density lipoprotein receptor class A domain containing 4
Anks1	–1.1719450	ankyrin repeat and SAM domain containing 1
Farp1	–1.1532675	FERM, ARH/RhoGEF and pleckstrin domain protein 1
Tenm4	–1.1483548	teneurin transmembrane protein 4
Ints4	–1.1287068	integrator complex subunit 4
Cped1	–1.1039643	cadherin–like and PC–esterase domain containing 1
Ubash3b	–1.0863720	ubiquitin associated and SH3 domain containing, B
Tmem184b	–1.0861458	transmembrane protein 184b
Cfh	–1.0712845	complement component factor h
Rad51b	–1.0331051	RAD51 paralog B
Nup153	–1.0255149	nucleoporin 153
Anapc1	–1.0234320	anaphase promoting complex subunit 1
Ireb2	–1.0130711	iron responsive element binding protein 2
Zfpm2	–1.0115828	zinc finger protein, multitype 2
Ctif	–1.0037613	CBP80/20–dependent translation initiation factor
Notch2	–1.0007407	notch 2

Gene Symbol	Log_2 Fold Change	Protein Name
Stk39	–0.9924127	serine/threonine kinase 39
Man2b1	–0.9859306	mannosidase 2, alpha B1
Ptprd	–0.9858860	protein tyrosine phosphatase receptor type D
Slc8a1	–0.9824997	solute carrier family 8 (sodium/calcium exchanger), member 1
Foxk2	–0.9776955	forkhead box K2
P4htm	–0.9763114	prolyl 4–hydroxylase, transmembrane (endoplasmic reticulum)
Aqr	–0.9760567	aquarius
Tmsb4x	–0.9729764	thymosin, beta 4, X chromosome
Ctdspl	–0.9710371	CTD small phosphatase like
Tnc	–0.9651683	tenascin C
Epha4	–0.9618025	Eph receptor A4
Cntln	–0.9612104	centlein, centrosomal protein
Stim2	–0.9596699	stromal interaction molecule 2
Dock4	–0.9570585	dedicator of cytokinesis 4
Tbc1d22a	–0.9560390	TBC1 domain family, member 22a
Atrnl1	–0.9526462	attractin like 1

Table S2. Upregulated genes.

Gene Symbol	Log_2 Fold Change	Protein Name
Grem1	2.4730930	gremlin 1, DAN family BMP antagonist
Ass1	1.9355703	argininosuccinate synthetase 1
Lhcgr	1.8762662	luteinizing hormone/choriogonadotropin receptor
Tox	1.7949206	thymocyte selection-associated high mobility group box
1110008P14Rik	1.7595323	
Gabrb2	1.7139991	gamma-aminobutyric acid type A receptor subunit beta 2
Ddit4	1.6753717	DNA-damage-inducible transcript 4
Mro	1.5897402	maestro
Rhox8	1.5796254	reproductive homeobox 8
Gstm7	1.5246381	glutathione S-transferase, mu 7
Olfm1	1.4962696	olfactomedin 1
Rnf13	1.4949999	ring finger protein 13
Hmgn3	1.4764976	high mobility group nucleosomal binding domain 3
Cpox	1.4594741	coproporphyrinogen oxidase
Gsta4	1.4164166	glutathione S-transferase, alpha 4
Tmem86a	1.3968822	transmembrane protein 86A
Cited2	1.3685162	Cbp/p300-interacting transactivator, with Glu/Asp-rich carboxy-terminal domain, 2
Cyp19a1	1.3513072	cytochrome P450, family 19, subfamily a, polypeptide 1
Etfbkmt	1.3401887	electron transfer flavoprotein beta subunit lysine methyltransferase
Dctn6	1.3260089	dynactin 6
Rnf114	1.2902892	ring finger protein 114
Skap2	1.2703797	src family associated phosphoprotein 2
Cbs	1.2585038	cystathionine beta-synthase
Smim10l2a	1.2563780	small integral membrane protein 10 like 2A
Klhl13	1.2538776	kelch-like 13
Smarcal1	1.2466536	SNF2 related chromatin remodeling ATPase like 1
Rnf25	1.2387927	ring finger protein 25
